# Pressure-Sensitive Walkway System for Evaluation of Lameness in Dogs Affected by Unilateral Cranial Cruciate Ligament Rupture Treated with Porous Tibial Tuberosity Advancement

**DOI:** 10.3390/vetsci10120696

**Published:** 2023-12-08

**Authors:** Marta Guadalupi, Alberto Maria Crovace, Donato Monopoli Forleo, Francesco Staffieri, Luca Lacitignola

**Affiliations:** 1 Dottorato di Ricerca in “Trapianti di Tessuti ed Organi e Terapie Cellulari”, Università degli Studi di Bari Aldo Moro, 70100 Bari, Italy; marta.guadalupi@uniba.it; 2 Dipartimento di Medicina di Precisione e Rigenerativa e Area Jonica, Università degli Studi di Bari Aldo Moro, 70100 Bari, Italy; francesco.staffieri@uniba.it; 3 Dipartimento di Medicina Veterinaria, Università di Sassari, 07100 Sassari, Italy; 4 Departamento de Ingeniería Mecánica, Instituto Tecnológico de Canarias (ITC), Añepa, esq. Tigotán s/n, 35118 Las Palmas de Gran Canaria, Spain; dmonopoli@itccanarias.org

**Keywords:** cranial cruciate ligament rupture, TTA, dog, gait analysis

## Abstract

**Simple Summary:**

The purpose of this study was to objectively evaluate the lameness of dogs with a unilateral cranial cruciate ligament rupture (CrCLR) treated with porous tibial tuberosity advancement before surgery and at three different timepoints after surgery. This was conducted using the GAITRite^®^ system, capable of calculating multiple spatiotemporal gait parameters simultaneously for each limb. Dogs presenting with hind limb lameness underwent a complete orthopedic examination. Patients with other orthopedic, neurologic, or neoplastic conditions were excluded from the study. Before surgery, the hind limb to be treated underwent radiographic examination and gait analysis. At least three similar gaits (same gait type and velocity) were recorded for each dog. In addition, all intraoperative and postoperative complications were considered. The lameness was assessed on the pressure-sensitive walkway before (T0) and 30 (T1), 90 (T2), and 120 (T3) days after surgery. Pressure measurements (gait lameness score and total pressure index percentage) were collected for S (treated with porous TTA) and C (healthy contralateral limb) at T0, T1, T2, and T3 and were statistically analyzed. An ANOVA test was performed to compare the data, and a value of *p* < 0.05 was considered significant. Twenty dogs (*n* = 20) of different breeds and ages with CrCLR were included in the study. The results showed that there was a statistically significant difference in the GAIT4Dog^®^ lameness score (GLS) and TPI% between S and C for each timepoint. Statistically significant differences in the GLS and TPI% between S at T0 and S at T2 and between S at T0 and S at T3 (*p* < 0.001) were found. The GLS and TPI% increased statistically significantly from 90 days after surgery compared to the preoperative measurements. Moreover, comparing the GLS and TPI% between the treated limb and the control limb showed that a statistically significant difference remained at each timepoint.

**Abstract:**

The aim of this study was to objectively evaluate lameness in dogs affected by a unilateral cranial cruciate ligament rupture (CrCLR) treated with porous tibial tuberosity advancement before surgery and at three different timepoints after surgery, using the GAITRite^®^ system (version 4.9Wr), a pressure-sensitive walkway system that is able to calculate several spatiotemporal gait parameters simultaneously for each limb. The dogs walked on the pressure-sensitive walkway before (T0) and 30 (T1), 90 (T2), and 120 (T3) days after surgery. Pressure measurements (gait lameness score and total pressure index %) were collected for S (treated with porous TTA) and C (healthy contralateral limb) at T0, T1, T2, and T3 and statistically evaluated. An ANOVA test was performed to compare the data, and a value of *p* < 0.05 was considered significant. Twenty dogs (n = 20) of various common breeds and ages with CrCLR were enrolled in the study. The results showed that there was a statistically significant difference in the GAIT4Dog^®^ lameness score (GLS) and TPI% between S and C for each timepoint. Statistically significant differences in the GLS and TPI% between S at T0 and S at T2 and between S at T0 and S at T3 (*p* < 0.001) were found. The results showed that there was a statistically significant difference in the GAIT4Dog^®^ lameness score (GLS) and TPI% between S and C for each timepoint. Statistically significant differences in the GLS and TPI% between S at T0 and S at T2 and between S at T0 and S at T3 were found. The GLS and TPI% increased statistically significantly from 90 days after surgery compared to the preoperative measurements. Moreover, comparing the GLS and TPI% between the treated limb and the control limb showed that a statistically significant difference remained at each timepoint.

## 1. Introduction

Cranial cruciate ligament rupture (CrCLR) is one of the most common pathologic conditions affecting the stifle joint in dogs [1]. This disorder has a significant negative impact on the patient’s quality of life, as it leads to stifle instability, resulting in hind limb lameness [2,3].

Several therapeutic options exist for the treatment of cranial cruciate ligament rupture, including conservative or surgical procedures, but surgery is considered the best approach in dogs weighing over 15 kg [4]. However surgical therapy was also proposed for small dogs [5]. Tibial Plateau levelling Osteotomy (TPLO) and tibial tuberosity advancement (TTA) are the most frequently performed surgical techniques in veterinary orthopedic surgery [6]. It has been demonstrated that TTA, which was first described in veterinary medicine in 2002 [7], performed using a porous titanium scaffold (porous TTA) is an optimal technique for surgical treatment of a stifle joint suffering from partial or complete CrCLR [8].

The clinical score alone is not sufficient to assess lameness, but the use of a more sensitive method is recommended [9]. Recently, in order to objectively assess the degree of lameness before and after surgery and to eliminate subjective influences that might affect clinical results, computer-based gait analysis systems to collect kinetic and kinematic parameters have become popular in veterinary medicine [10].

To perform a kinetic analysis, the most-used device is the force plate platform, through which it is possible to record the ground reaction forces (GRFs). The gold standard for objectively measuring lameness in dogs with orthopedic diseases is considered the gait analysis performed by a force plate platform [9,11]. The force plate platform is used to assess balance or swing indirectly by recording the force the body applies to the ground. From this measurement, the center of pressure (CoP) can be determined using pressure or force transducers; these devices provide an electrical signal proportional to the force applied. Strain gauges, piezoelectric, piezoresistive, and capacitive transducers are a few different types of force or pressure transducers. A standard force plate platform consists of a flat top plate supported by three or four force or pressure transducers. The relative forces perceived by each of these angular transducers result in the CoP position of a body [12]. However, this system is characterized by several disadvantages, such as difficulties in collecting data simultaneously from all four limbs during one cycle without an associated treadmill [13]; lack of information on the function of the limb during the swing phase of the stride [14]; the necessity of a complex installation on the flat surface; and the inability to move the system easily. For all of these reasons, the pressure-sensitive walkway (PSW) system has been validated as a successful alternative to the force platform [15]. The PSW has sensors integrated into rigid platforms to produce pressure platform matrices. By collecting data on plantar pressure distribution, the entire plantar surface can be evaluated. Furthermore, as in the case of force plate platforms, through the summation of all weight sensors, the vertical component of the GRFs and thus the position of the CoP can be obtained. Due to sensor size and spatial resolution (number of sensors), the obtained CoP may be less accurate than that obtained from the force plate platforms [16]. Pressure platforms also measure spatiotemporal gait characteristics, providing a kinematic study of gait [17,18].

In general, the PSW is likely the best option to obtain valid data on load distribution, load transfers, and pressure distribution, while the force plate platform is the best choice to obtain an evaluation of balance through the high accuracy of CoP data.

The aim of this study is to evaluate lameness in dogs affected by unilateral CrCLR treated with porous TTA before surgery, at three different timepoints after surgery, in order to verify that this technique is an effective method that improves load on the affected hind limb. To objectively perform the load assessments, we used the GAITRite^®^ system, a portable pressure walkway system combined with the original software (version 4.9Wr) developed for quadrupeds (GAITFour^®^), which can calculate pressure measurements and several spatiotemporal gait parameters simultaneously for each limb. By using the GAITRite^®^ walkway system to detect gait alterations, an objective evaluation of lameness more sensitive than the visual score could be obtained in order to provide significant data to monitor the recovery of clinical function in hind limbs affected by unilateral CrCLR treated with porous TTA.

The validity of this system has been amply demonstrated in human medicine, and it is currently widely used for the diagnosis of musculoskeletal disorders [19] or for Parkinson’s gait analysis [20]. In veterinary medicine, the PSW has been used to perform a kinetic assessment of normal walking and jumping in cats [21], to measure changes in the location of the center of pressure and hoof-unrollment pattern in relation to an 8-week shoeing interval in horses [22], to quantify lameness in pigs with induced osteoarthritis [23], to verify the effects of trimming on dairy cattle hoof [24], or to determine the validity of conservative treatment in dogs with pelvic fractures [25]. Furthermore, even in veterinary medicine, it has been recently proven that the pressure-sensitive walkway provides a more accurate degree of lameness than the visual score alone [26].

Our hypothesis was that in dogs with CrCLR, the values of preoperative pressure measurements of the affected hind limb would be significantly lower than those of the contralateral hind limb, and we also expected to obtain a significant improvement in the postoperative values of pressure measurements of the surgically treated hind limb.

## 2. Materials and Methods

### 2.1. Animals

Dogs presenting with hind limb lameness at the surgical clinic department (DiMePRe-J) of the University of Bari Aldo Moro, between January 2021 and January 2022, underwent a full orthopedic examination. Unilateral complete CrCLR was diagnosed by performing the cranial drawer test, the tibial compression test, and the sitting test. Patients with clinical signs or a history of other orthopedic, neurological, neoplastic diseases, or meniscal diseases of the limb affected by unilateral complete CrCLR were excluded from the study.

All of the dogs that presented with any disease on the contralateral limb or needed to be treated with an anti-inflammatory drug throughout the follow-up period considered for this study were excluded from the assessment.

The hind limb to be treated was radiographically evaluated to investigate indirect signs of CrCLR.

For each patient, the breed, weight, age, sex, and BCS were recorded. BCS was classified according to a previous scale (range from 1 to 9) [27].

Ethics committee approval was not required for this study, as it was a clinical study conducted after the owner had signed informed consent.

### 2.2. Surgical Procedure

**Figure 1:**
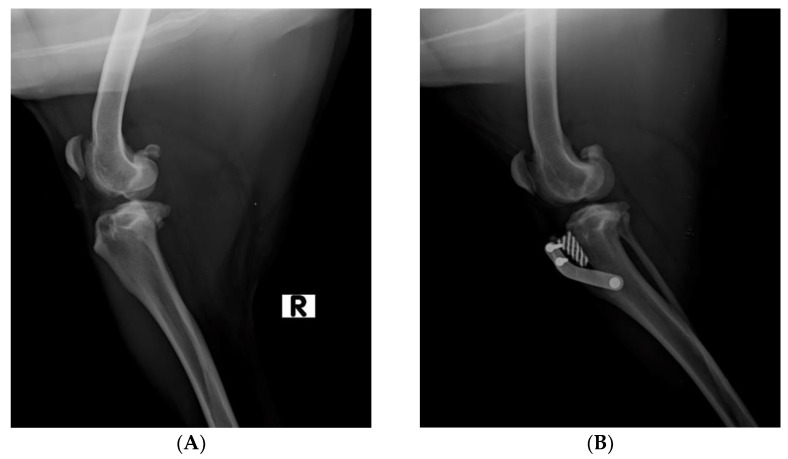
(**A**). Pre-operative X-ray examination in mediolateral view of dog’s stifle. (R: right limb) (**B**). Post-operative X-ray examination in mediolateral view of dog’s stifle.

Prior to surgery, an X-ray examination was performed in mediolateral views and with flexion as close as possible to 135 degrees, in order to estimate the advancement of the tibial tuberosity (Figure 1A).

The dogs included in the study underwent general anesthesia. Surgery was performed under totally aseptic conditions. Prior to performing the porous TTA, the stifle joint was arthroscopically investigated to assess any meniscal disease or cartilage damage and validate the clinical diagnosis of a complete or partial rupture of the CrCL. According to the inclusion criteria, only dogs affected by complete CrCLR were enrolled in the current study. Once the arthroscopic procedure was completed, the porous TTA surgery was performed. A kit containing all of the necessary instrumentation, which was made by the ICT of Canaries, was used to perform the procedure. First, a Maquet’s hole on the medial side of the distal tibial tuberosity was performed. Thereafter, the tibial tuberosity was osteotomized using a special guide, and a distractor was used to gradually advance the osteotomy to enable the insertion of a porous titanium scaffold. A titanium plate with three screws was placed on the cranial portion of the osteotomized tibial tuberosity, and the tibial diaphysis and the surgical site was sutured. At the end of the surgical procedure, an X-ray examination was performed to assess the correct advancement of the tibial tuberosity [8] (Figure 1B).

The same experienced orthopedic surgeons (LL and AMC) performed the surgical procedure.

In addition, any intraoperative (fracture/fissure of the tibial tuberosity through the Maquet’s hole) and postoperative complications were classified according to a previously described definition [28] as minor, where additional surgical or medical treatment to resolve (seroma of the surgical wound) the problem was not required, or major, which required surgical or medical treatment to resolve (i.e., postoperative tibial fracture, surgical wound dehiscence, or plate cracks) the issue. Patients with major postoperative complications that required any surgical or medical treatment to resolve were excluded from the study.

Prior to gait analysis, a complete radiographic examination was performed for each patient in the mediolateral and craniocaudal views at each timepoint considered, in order to exclude any major orthopedic complications and to verify proper bone healing.

### 2.3. Gait Analysis

**Figure 2:**
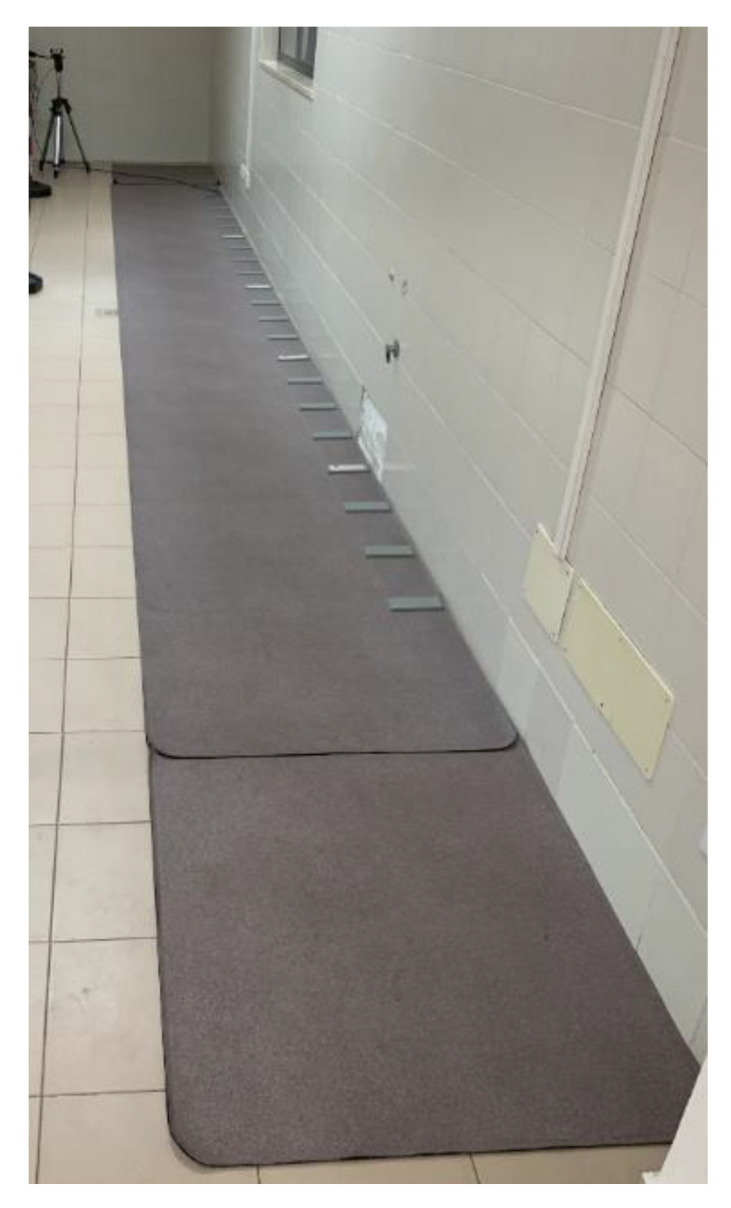
GAITFour^®^Dog pressure-sensitive walkway used to perform this study. The PSW is equipped with encapsulated sensor pads available along the entire length of the mat. On each side of the PSW, cameras are positioned at a height of 50 cm. To identify the start and end points of the walk, an inactive mat was placed at each end of the walkway system.

This study was performed using the GAITRite^®^ system, which consisted of a pressure-sensitive walkway (GAIT4 Dog^®^ walkway, CIR Systems Inc., Sparta, NJ, USA, Figure 2) and a dedicated software product developed for quadrupeds (GAITFour^®^ software version 4.9Wr, CIR Systems Inc., Sparta, NJ, USA).

The PSW is equipped with encapsulated sensor pads. Each sensor pad has an active area of 24 inches squared (61 cm squared) and contains 2304 sensors arranged in a 48 × 48 grid pattern. The sensors are placed on 0.5-inch (1.27 cm) centers. Multiple sensor pads are connected to form the desired length of the walkway. In addition, to identify the start and end point of the walk, a 1.25 × 0.85 m section of inactive mat was placed at each end of the walkway system. For each trial, the corresponding video was collected by cameras (Logitech mega pixel Web camera, Logitech, Fremont, Calif.; Phillips pixel plus Web camera, Philips Electronics North America Corp, New York, NY, USA) placed on each side of the PSW at a height of 50 cm. The video clips were automatically linked to the patient’s data files. Once the trials were completed, one author (MG) examined videos of each pass to ensure that the inclusion criteria were met.

At the end of the data analysis, a complete report was obtained for each patient, and this was exported to a spreadsheet (Microsoft Office Excel 2019, Microsoft Corp, Redmond, Washington, DC, USA).

Pressure measurements (GAIT4Dog^®^ Lameness Score and Total Pressure Index %) and spatiotemporal parameters (velocity, stride length, stance time, stance % of cycles, swing % of cycles, swing time, and number of sensors) were considered for this investigation and were collected for hind limbs affected by unilateral CrCLR treated with porous TTA (treated limb) and healthy contralateral hind limbs, in order to provide a control evaluation (control limb).

The gait analysis was performed at four different timepoints: before surgery (T0) and at 30 (T1), 90 (T2), and 120 (T3) days after surgery.

**Figure 3:**
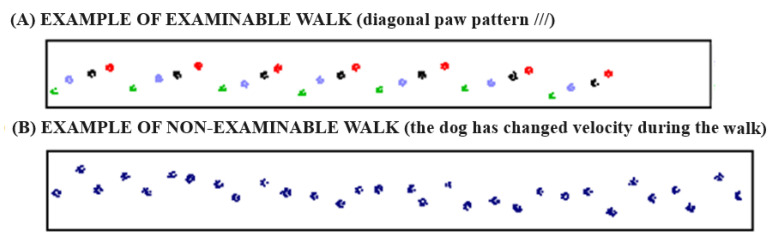
Two different patterns of walk: (**A**) is an example of a valid walk with regular gait cycles (same gait and velocity), which can be processed to collect data; (**B**) is an example of a not valid walk with irregular gait cycles (different gait and velocity during the walk), which is unable to be processed.

Each dog was led down the walkway by the same handler. The trials were performed in a quiet and isolated environment so there were no distractions for the dog, which could influence the analysis. To standardize the procedure, data were collected using the walk as the gait. Throughout the trials, the dogs had to walk along the center of the mat with a loose leash and straight head. The walk was performed continuously in both directions. To provide an objective assessment of lameness, a minimum of three similar walks (same gait and velocity) with a minimum of three gait cycles were collected for each dog. To be analyzed, the walkway had to show a regular walking pattern and a consistent velocity with a variability of less than 10% (Figure 3).

#### 2.3.1. Pressure Measurements

The GAIT4 Dog^®^ lameness score (GLS) is a specific parameter that identifies a decreased load on a limb as an indication of pain during the stance phase. Thus, the GLS is a scale that describes the degree of offloading (lameness) and overloading (compensation) of an animal’s limb. A score of 100 indicates the absence of lameness, while scores above 108 indicate compensation applied by the animal on the contralateral leg. In particular, limb GLS values below 100 indicate lameness (the lower the value, the greater the degree of lameness of the limb in question), while limb GLS values above 100 indicate overload (the higher the value, the greater the degree of compensation of the limb in question). While the animal is walking on the walkway, the system collects the variables derived from a combination of pressure data, time data, and position data.

The total pressure index percentage (TPI%) indicates the percentage of weight distribution on each limb (the total should be 100%, 30% for each forelimb and 20% for each hind limb).

#### 2.3.2. Spatiotemporal Parameters

The spatiotemporal parameters that are recorded include velocity (obtained after dividing the distance traveled by the ambulation time), stride length (measured between the heel points of two consecutive impressions of the same paw), number of sensors (number of sensors activated by the contact of each paw), stance time (the weight-bearing part of the gait cycle, indicating the elapsed time between the first contact and the last contact of an identified paw), swing time (the non-weight-bearing part of each gait cycle, indicating the elapsed time between the last contact and the first contact of an identified paw), stance % of cycles (the percentage of the stance time relative to the gait cycle), and swing % of cycles (the percentage of the swing time relative to the gait cycle).

### 2.4. Statistical Analysis

The data were collected and statistically evaluated with statistical software (The jamovi project (2022). jamovi. (Version 2.3) [computer software]. Retrieved from https://www.jamovi.org, access date 3 January 2023). The normal distribution was assessed using the Kolmogorov–Smirnov test. The data were analyzed using a two-way analysis of variance (ANOVA) for normally distributed data, followed by a post hoc Tukey HSD test. The analysis was conducted a priori on a pilot sample before starting the study. All data are expressed as the mean ± standard deviation (SD). *p*-values of <0.05 were considered significant.

## 3. Results

### 3.1. Animals

A total of twenty (n = 20) dogs met the inclusion criteria, among which one (n = 1) was a Saint Bernard, two (n = 2) were American pit bull terriers, one (n = 1) was a German Shepherd, one (n = 1) was an English pointer, one (n = 1) was a Tosa Inu, one (n = 1) was a Dogue de Bordeaux, one (n = 1) was a Boxer, three (n = 3) were beagles, one (n = 1) was an American Staffordshire terrier, two (n = 2) were Corsican dogs, one (n = 1) was a Labrador retriever, two (n = 2) were Rottweilers, and three (n = 3) were mixed-breed dogs. Fourteen (n = 14) dogs were females and six (n = 6) dogs were males. The mean age was 87.6 ± 40.6 months, the mean weight was 35.1 ± 14.77 kg, and the BCS was 5.4 ± 0.940 (Table 1). Nine (n = 9) of the dogs enrolled had CrCLR of the left hind limb and eleven (n = 11) had CrCLR of the right hind limb.

No intraoperative or major postoperative complications were recorded. Minor postoperative complications were recorded in five patients (n = 5), i.e., a wound seroma that resolved spontaneously in all cases.

### 3.2. Gait Analysis

A mean of 5.3 walkways (passages of the dogs from the starting point to the stopping point of the mat) of the mat collected per patient was excluded from the analysis. The most frequent cause of exclusion was a change in the average cycle stride velocity of >10%. Other less frequent causes of exclusion were walking too close to the sensors or handling with a highly tensioned leash, which causes a reduction in load on the forelegs, distorting the investigation.

**Figure 4:**
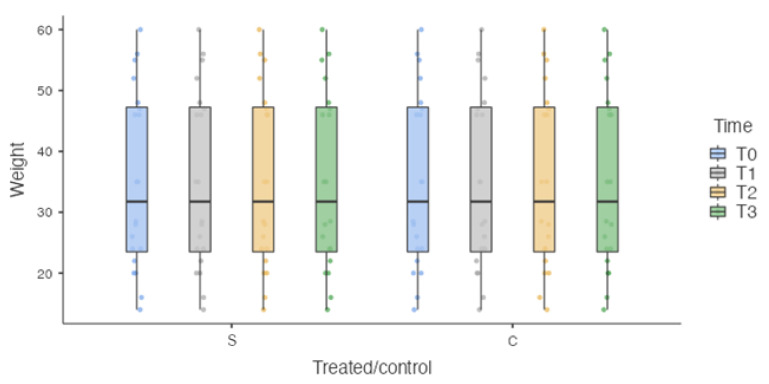
Box plot. S: surgically treated limbs. C: healthy limbs. No Statistically significant differences were detected at each time point for weight.

**Figure 5:**
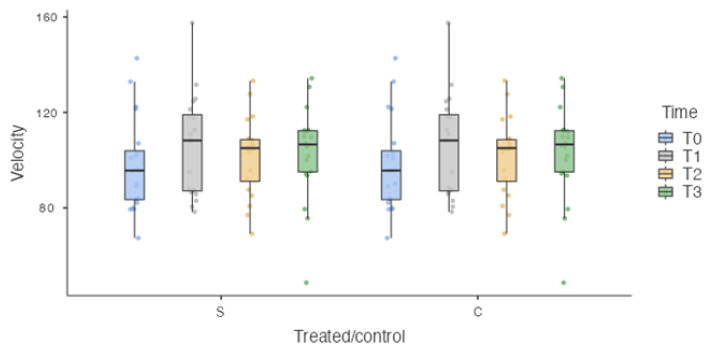
Box plot. S: surgically treated limbs. C: healthy limbs. No Statistically significant differences were detected at each time point for velocity.

The sample was homogeneously distributed in relation to the unbalanced independent variables, such as weight (35.1 ± 14.77 kg) (Figure 4) and velocity (102.131 ± 19.09 cm/s) (Figure 5).

### 3.3. Pressure Measurements

**Figure 6:**
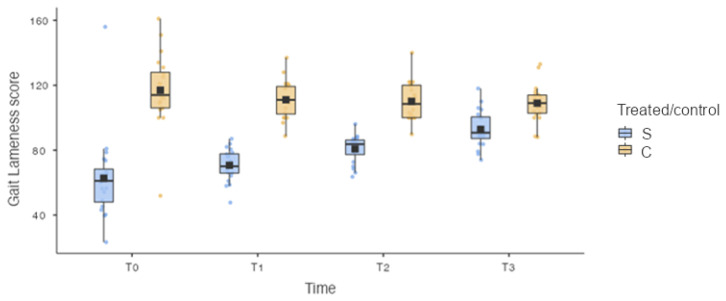
Box plot. S: treated limb; C: contralateral limb. Statistically significant difference was observed for the GAIT4Dog^®^ lameness score (GLS) between the treated limb and the control limb for each timepoint. Statistically significant differences in the GLS between the treated limb (S) at T0 and the treated limb (S) at T2 (*p* = 0.007) and between the treated limb (S) at T0 and the treated limb (S) at T3 (*p* < 0.001) were found.

Analysis of the results showed a statistically significant difference in the GAIT4Dog^®^ lameness score (GLS) between the treated limb (S, treated limb) and the control limb (C, contralateral limb) for each timepoint: 62.7 ± 26.35 for T and 117.0 ± 22.61 for C at T0 (*p* < 0.001); 70.7 ± 9.89 for T and 111.1 ± 11.50 for C at T1 (*p* < 0.001); 80.8 ± 8.53 for T and 110.1 ± 12.04 for C at T2 (*p* < 0.001); and 92.8 ± 11.22 for T and 109.0 ± 11.29 for C at T3 (*p* = 0.025) (Figure 6).

**Figure 7:**
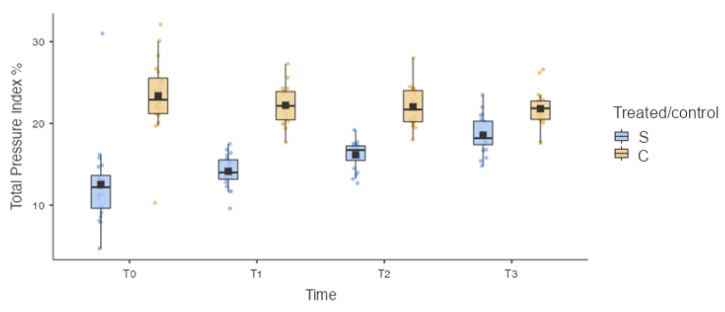
Box plot. S: treated limb; C: contralateral limb. Statistically significant difference was recorded for the total pressure index % (TPI%) between the treated limb (S) and the control limb (C) for each timepoint. Statistically significant differences in the TPI% between the treated limb (S) at T0 and the treated limb (S) at T2 (*p* = 0.007) and between the treated limb (S) at T0 and the treated limb (S) at T3 (*p* < 0.001) were found.

Similarly, a statistically significant difference was recorded for the total pressure index % (TPI%) between the treated limb (S) and the control limb (C) for each timepoint: 12.5 ± 5.23 for S and 23.4 ± 4.52 for C at T0 (*p* < 0.001); 14.2 ± 1.97 for S and 22.2 ± 2.30 for C at T1 (*p* < 0.001); 16.2 ± 1.71 for S and 22.0 ± 2.42 for C at T2 (*p* < 0.001); and 18.6 ± 2.25 for S and 21.8 ± 2.25 for C at T3 (*p* = 0.025) (Figure 7).

Moreover, the statistical analysis did not show a statistically significant difference for GLS between the treated limb (S) at T0 and the treated limb (S) at T1 (*p* = 0.723). However, statistically significant differences in the GLS between the treated limb (S) at T0 and the treated limb (S) at T2 (*p* = 0.007) and between the treated limb (S) at T0 and the treated limb (S) at T3 (*p* < 0.001) were found (Figure 6).

Similarly, the statistical analysis did not show a statistically significant difference in the TPI% between the treated limb (S) at T0 and the treated limb (S) at T1 (*p* = 0.714), and statistically significant differences in the TPI% between the treated limb (S) at T0 and the treated limb (S) at T2 (*p* = 0.007) and between the treated limb (S) at T0 and the treated limb (S) at T3 (*p* < 0.001) were found (Figure 7).

### 3.4. Spatiotemporal Parameters

**Figure 8:**
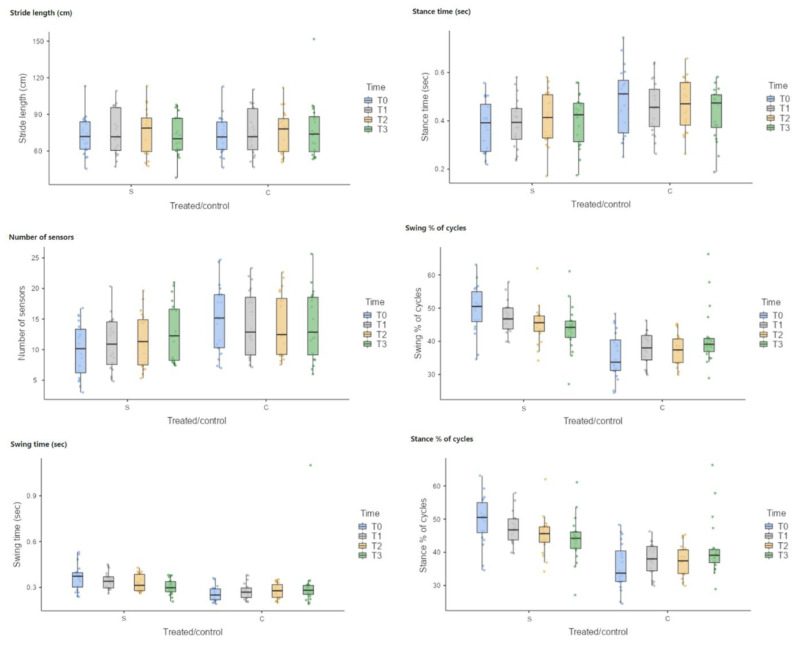
Box plot. For number of sensors (NS), statistically significant differences were observed between the treated limb (S) and the control limb (C) at T0. For the swing time (SwT), statistically significant differences were observed between the treated limb (S) and the control limb (C) at T0. Concerning the swing % of cycles (Sw%), statistically significant differences were observed between the treated limb (S) and the control limb (C) at T0, T1, and T2. Similarly, for the stance % of cycles (St%), statistically significant differences were observed between the treated limb (S) and the control limb (C) at T0, T1, and T2.

Statistical analysis of the data revealed no statistically significant difference in the stride length (SrL) between the treated limb (S) and the control limb (C) for each timepoint: 72.567 ± 15.7652 for S and 72.509 ± 16.0627 for C at T0 (*p* = 1.000); 75.299 ± 18.5031 for S and 75.408 ± 18.9477 for C at T1 (*p* = 1.000); 75.115 ± 18.9493 for S and 74.963 ± 17.7042 for C at T2 (*p* = 1.000); and 72.499 ± 16.8979 for S and 77.039 ± 23.3812 for C at T3 (*p* = 0.994) (Figure 8).

Moreover, there were no statistically significant differences for the stance time (ST) between the treated limb (S) and control limb (C) for each timepoint: 0.377 ± 0.1056 for S and 0.483 ± 0.1395 for C at T0 (*p* = 0.059); 0.390 ± 0.1017 for S and 0.458 ± 0.1084 for C at T1 (*p* = 0.527); 0.410 ± 0.1092 for S and 0.467 ± 0.1097 for C at T2 (*p* = 0.750); and 0.402 ± 0.1069 for S and 0.435 ± 0.1070 for C at T3 (*p* = 0.984) (Figure 8).

Concerning the number of sensors (NS), statistically significant differences were observed between the treated limb (S) and the control limb (C) at T0 (S-T0 = 9.941 ± 4.3440, C-T0 = 15.070 ± 5.4859, *p* = 0.028), whereas no statistically significant differences were observed between the treated limb (S) and the control limb (C) at T1 (S-T1 = 10.965 ± 4.3131, C-T1 = 14.227 ± 5.4303, *p* = 0.429); at T2 (S-T2 = 11.692 ± 4.4036, C-T2 = 14.303 ± 5.0718, *p* = 0.706); and at T3 (S-T3 = 12.910 ± 4.8358, C-T3 = 13.986 ± 5.4854, *p* = 0.997) (Figure 8).

Similarly for the swing time (SwT), statistically significant differences were observed between the treated limb (S) and the control limb (C) at T0 (S-T0 = 0.366 ± 0.0832, C-T0 0.258 ± 0.0494, *p* < 0.003), whereas no statistically significant differences were observed between the treated limb (S) and the control limb (C) at T1 (S-T1 = 0.342 ± 0.0547, C-T1 0.275 ± 0.0530, *p* = 0.195); at T2 (S-T2 = 0.328 ± 0.0541, C-T2 0.275 ± 0.0499, *p* = 0.531); and at T3 (S-T3 = 0.304 ± 0.0501, C-T3 0.317 ± 0.1896, *p* = 1,000) (Figure 8).

Concerning the swing % of cycles (Sw%), statistically significant differences were observed between the treated limb (S) and the control limb (C) at T0 (S-T0 = 49.655 ± 7.2122, C-T0 35.690 ± 7.0123, *p* < 0.001); at T1 (S-T1 = 47.280 ± 4.6900, C-T1 37.860 ± 4.8301, *p* < 0.001); and at T2 (S-T2 = 45.145 ± 5.7554, C-T2 37.490 ± 4.5966, *p* < 0.005), whereas no statistically significant differences were observed between the treated limb (S) and the control limb (C) at T3 (S-T3 = 43.875 ± 6.9425, C-T3 41.115 ± 8.6029, *p* = 0.868) (Figure 8).

Similarly, for the stance % of cycles (St%), statistically significant differences were observed between the treated limb (S) and the control limb (C) at T0 (S-T0 = 49.655 ± 7.2122, C-T0 35.690 ± 7.0123, *p* < 0.001); at T1(S-T1 = 47.280 ± 4.6900, C-T1 = 37.860 ± 4.8301, *p* < 0.001); and at T2 (S-T2 = 45.145 ± 5.7554, C-T2 = 37.490 ± 4.5966, *p* = 0.005), whereas no statistically significant differences were observed between the treated limb (S) and the control limb (C) at T3 (S-T3 = 43.875 ± 6.9425, C-T3 = 41.115 ± 8.6029, *p* = 0.866) (Figure 8).

## 4. Discussion

The PSW is a valid system for objectively assessing lameness [29], eliminating from the analysis any bias resulting from the subjectivity of the evaluation based only on observing the animal walking [15]. Specifically, this system assesses limb positioning, overloading, offloading, and the restriction of movement performed by precise and simple parameters, in contrast to clinical scoring, which is based on the use of simple descriptions of optical observations by different operators. Moreover, the subjective assessment of gait must be interpreted with caution due to, for example, the different experience of the operators or the poor inter- and intra-observer agreement [9].

In our study, our initial hypothesis was satisfied that in dogs with CrCLR, the values of preoperative GLS and TPI% of the affected hind limb were significantly lower than those of the contralateral hind limb, and we obtained a significant improvement in the postoperative values of GLS and TPI% of the surgically treated hind limb.

In this study, the software GAITFour^®^ was able to record the pressure measurements and spatiotemporal parameters simultaneously for each paw for the gaits of dogs of different sizes while walking [30].

The protocol of this study involved the recording of the pressure measurements GAIT4 Dog^®^ lameness score (GLS) [31] and total pressure index % (TPI%) [32], for quantitative detection of lameness. Also, spatiotemporal parameters such as velocity, stride length, number of sensors, stance time, swing time, stance % of cycles, and swing % of cycles were collected [33].

Trials were conducted by walking the dogs in both directions of the PSW, as it was shown that the results were not influenced by the direction of walking on the walkway [34].

Since velocity and weight are considered to be unbalanced independent variables with a negative influence on the objective assessment of spatiotemporal parameters [35], the gait analysis was conducted at a walk to standardize the procedure and to obtain as homogeneous an average velocity as possible. The weight of the dogs was homogeneous, as the surgical procedure was only performed on medium to large dogs. Therefore, the analysis can be considered bias-free, as the sample was homogeneous in relation to these two variables. In addition, the use of walking as the speed at which to conduct the trials is based on the reason that in more severe cases of lameness, the dog’s discomfort increases with trotting, resulting in the subtraction of the load during the stance phase [36]. An inability to collect results from dogs with severe lameness would result in the loss of these data, reducing the power of the study [37].

To improve the objectivity of the evaluation of the recovery of the hind limb treated with porous TTA, the study protocol considers the contralateral healthy hind limb as a control. Therefore, it was possible to exclude any influences that the use of dogs with different weights and conformations than those included in the study as the control may have provided. Since hind limb lameness compensation is carried out by the contralateral hind limb, pressure measurements and spatiotemporal parameters of the forelimbs were not collected in this study [38].

Regarding the pressure measurements that were considered in this study, it was observed that the GLS and TPI% increased statistically significantly from 90 days after surgery compared to the preoperative measurements. The degree of lameness improved significantly from 90 days post-surgery, as complete osseointegration is appreciated radiographically at 90 days post-surgery. In agreement with other studies [39,40], radiographic examinations performed at T1 revealed the early signs of bone deposition at the osteotomy site, while complete bone healing was observed at T2.

**Figure 9:**
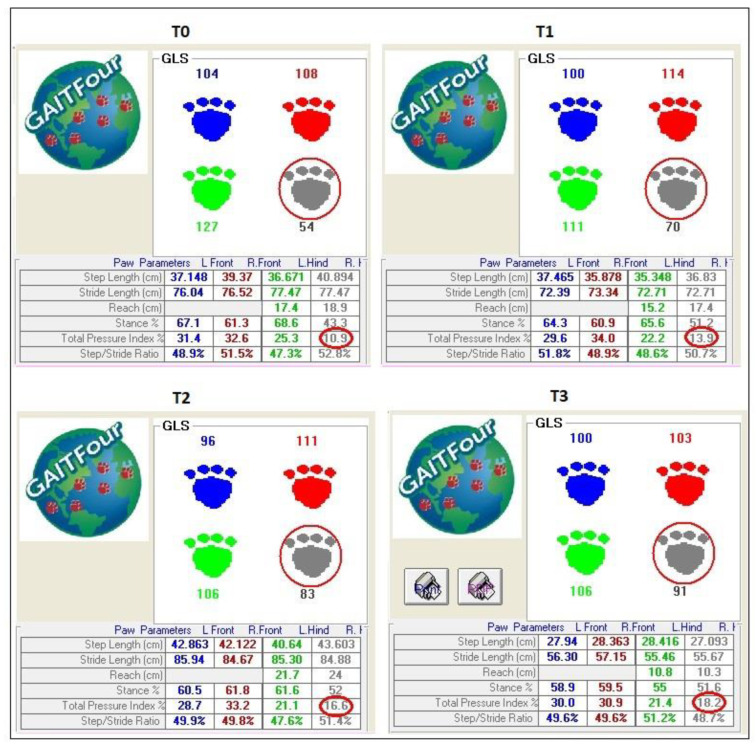
GLS and TPI% at different timepoints (T0, T1, T2, T3). GLS and TPI% increase gradually in each timepoint, with a significant difference between pre-operative and postoperative measurements from 90 days onwards. However, the difference between the treated limb and the control limb remains significant for each timepoint considered in the study, showing the overload on the healthy contralateral limb. The red circles show the lame limb.

Moreover, comparing the GLS and TPI% between the treated limb and the control limb showed that a statistically significant difference remained at each timepoint. These results suggest that there is an increased load on the affected limb, but despite this, some degree of lameness persists after surgery, as the difference between the treated and control limbs is significant until the last timepoint assessed in this study. These results suggest that the overload on the contralateral healthy limb remains even 120 days after surgery (Figure 9).

The investigation of the spatiotemporal parameters showed no significant difference between the treated and the control limb in each of the timepoints examined in this study, whereas a significant improvement was found in the number of sensors [41] activated during walking and the swing time from 30 days post-surgery and the swing % of cycles and stance % of cycles from 90 days post-surgery. Therefore, statistical analysis of spatiotemporal parameters (NS, SwT, Sw%, and St%) suggests a statistically significant improvement in loading, with a consequent reduction in pain of limbs treated with porous TTA.

In a study in which limbs with unilateral CrCLR were treated performing the modified Maquet procedure (MMP), significant improvement in the GRF was observed from 30 to 90 days post-surgery, while at 90 days, a significant difference was recorded in the PVF and VI between the treated and healthy limbs. These results were obtained using a force plate [39]. Furthermore, in another study in which force plate gait analysis was performed to evaluate the function of the limb treated with tibial tuberosity advancement (TTA) in dogs affected by unilateral CrCLR, there was a significant increase in the VF from 4 to 16 months after surgery in the treated limbs compared to before surgery, but these forces were significantly lower than in the healthy dogs used as controls [42]. In both studies, it was reported that both surgical techniques performed resulted in a significant improvement in limb function compared to preoperative values, but the VF remained significantly lower in all of the treated limbs compared to the control limb. Despite the different methodologies used, the results obtained in both studies agree with the results of our study.

A possible limitation of this study could be the inability of the pressure-sensitive walkway system to measure forces in the three dimensions (vertical, mediolateral, and craniocaudal forces), providing information only on vertical forces [43]; however, a previous study showed that vertical force variables had better accuracy than craniocaudal force variables in diagnosing hind limb lameness in dogs [44].

Therefore, the main limitations of this study included the small sample size, the short follow-up period, the different breeds of dogs included in the study, the time elapsed between cruciate rupture and surgery, and the observance of postoperative exercise restriction of the dogs by the owners. Moreover, although the patellar tendon-tibial plateau angles (PL-TPA) could affect clinical outcomes [45], they were not included in our analysis. Another limitation of this study is that the presence and degree of osteoarthritis at the time of surgery were not considered. Certainly, how osteoarthritis could influence the functional recovery of the joint after surgery should be investigated, but it was not the purpose of the current study.

## 5. Conclusions

In conclusion, dogs with CrCLR treated with porous TTA showed functional recovery of the stifle from 90 days post-surgery, with an objective increase in loading in the medium term. Moreover, the overload on the healthy contralateral limb was reduced at 120 days post-surgery, but not significantly, as a certain degree of offload on the treated limb and, consequently, a certain degree of overload on the healthy limb was maintained. Therefore, porous TTA represents a viable surgical alternative among all the surgical options described in veterinary orthopedics, involving a positive outcome on patients’ quality of life.

## Figures and Tables

**Table 1:** Mean ± SD of BCS, age, and weight.

	BCS	AGE (Months)	WEIGHT (kg)
N	20	20	20
Mean	5.40	87.6	35.1
Median	5.00	78.0	31.8
SD	0.940	40.7	14.77
Minimum	4.00	24.0	14.0
Maximum	8.00	156	60.0

## Data Availability

Data are contained within the article.

## References

[1] Jerram R.M., Walker A.M. (2003). Cranial cruciate ligament injury in the dog: Pathophysiology, diagnosis and treatment. N. Z. Vet. J..

[2] Spinella G., Arcamone G., Valentini S. (2021). Cranial Cruciate Ligament Rupture in Dogs: Review on Biomechanics, Etiopathogenetic Factors and Rehabilitation. Vet. Sci..

[3] Wucherer K.L., Conzemius M.G., Evans R., Wilke V.L. (2013). Short-term and long-term outcomes for overweight dogs with cranial cruciate ligament rupture treated surgically or nonsurgically. J. Am. Vet. Med. Assoc..

[4] Innes J.F., Bacon D., Lynch C., Pollard A. (2000). Long-term outcome of surgery for dogs with cranial cruciate ligament deficiency. Vet. Rec..

[5] Amimoto H., Koreeda T., Ochi Y., Kimura R., Akiyoshi H., Nishida H., Miyabayashi T., Beale B.S., Hayashi K., Wada N. (2020). Force Plate Gait Analysis and Clinical Results after Tibial Plateau Levelling Osteotomy for Cranial Cruciate Ligament Rupture in Small Breed Dogs. Vet. Comp. Orthop. Traumatol..

[6] von Pfeil D.J.F., Kowaleski M.P., Glassman M., Dejardin L.M. (2018). Results of a survey of Veterinary Orthopedic Society members on the preferred method for treating cranial cruciate ligament rupture in dogs weighing more than 15 kilograms (33 pounds). J. Am. Vet. Med. Assoc..

[7] Montavon P.M., Damur D.M., Tepic S. Advancement of the tibial tuberosity for the treatment of the cranial cruciate deficient stifle. Proceedings of the 1st World Orthopaedic Veterinary Congress.

[8] Trisciuzzi R., Fracassi L., Martin H.A., Monopoli Forleo D., Amat D., Santos-Ruiz L., De Palma E., Crovace A.M. (2019). 41 Cases of Treatment of Cranial Cruciate Ligament Rupture with Porous TTA: Three Years of Follow Up. Vet. Sci..

[9] Waxman A.S., Robinson D.A., Evans R.B., Hulse D.A., Innes J.F., Conzemius M.G. (2008). Relationship between objective and subjective assessment of limb function in normal dogs with an experimentally induced lameness. Vet. Surg..

[10] Böddeker J., Drüen S., Meyer-Lindenberg A., Fehr M., Nolte I., Wefstaedt P. (2012). Computer-assisted gait analysis of the dog: Comparison of two surgical techniques for the ruptured cranial cruciate ligament. Vet. Comp. Orthop. Traumatol..

[11] Quinn M.M., Keuler N.S., Lu Y., Faria M.L., Muir P., Markel M.D. (2007). Evaluation of agreement between numerical rating scales, visual analogue scoring scales, and force plate gait analysis in dogs. Vet. Surg..

[12] Jacinta B., Neil O.H. (2000). A quality control procedure for force platforms. Physiol. Meas..

[13] Brebner N.S., Moens N.M., Runciman J.R. (2006). Evaluation of a treadmill with integrated force plates for kinetic gait analysis of sound and lame dogs at a trot. Vet. Comp. Orthop. Traumatol..

[14] Bertram J.E.A., Lee D.V., Todhunter R.J., Foels W.S., Jo Williams A., Lust G. (1997). Multiple Force Platform Analysis of the Canine Trot: A New Approach to Assessing Basic Characteristics of Locomotion. Vet. Comp. Orthop. Traumatol..

[15] LeQuang T., Maitre P., Roger T., Viguier E. (2010). Is a Pressure Walkway System Able to Highlight a Lameness in Dog?. Proceedings of the 6th World Congress of Biomechanics (WCB 2010).

[16] Guldemond N. (2007). Plantar pressure measurement. Plantar Pressure, Diabetes and Amputation: Studies on Etiological, Diagnostic and Therapeutical Aspects.

[17] Kim J., Kazmierczak K.A., Breur G.J. (2011). Comparison of temporospatial and kinetic variables of walking in small and large dogs on a pressure-sensing walkway. Am. J. Vet. Res..

[18] Sandberg G., Torres B., Berjeski A., Budsberg S. (2018). Comparison of Simultaneously Collected Kinetic Data with Force Plates and a Pressure Walkway. Vet. Comp. Orthop. Traumatol..

[19] Błaszczyk J.W. (2016). The use of force-plate posturography in the assessment of postural instability. Gait Posture.

[20] Nelson A.J., Zwick D., Brody S., Doran C., Pulver L., Rooz G., Sadownick M., Nelson R., Rothman J. (2002). The validity of the GaitRite and the Functional Ambulation Performance scoring system in the analysis of Parkinson gait. NeuroRehabilitation.

[21] Lascelles B.D., Findley K., Correa M., Marcellin-Little D., Roe S. (2007). Kinetic evaluation of normal walking and jumping in cats, using a pressure-sensitive walkway. Vet. Rec..

[22] Van Heel M.C., Moleman M., Barneveld A., Van Weeren P.R., Back W. (2005). Changes in location of centre of pressure and hoof-unrollment pattern in relation to an 8-week shoeing interval in the horse. Equine Vet. J..

[23] Uilenreef J., van der Staay F.J., Meijer E. (2019). A Monosodium Iodoacetate Osteoarthritis Lameness Model in Growing Pigs. Animals.

[24] Carvalho V., Nääs I.A., Bucklin R.A., Shearer J.K., Shearer L., Massafera V., Souza S.R.L.d. (2006). Effects of trimming on dairy cattle hoof weight bearing surfaces and pressure distributions. Braz. J. Vet. Res. Anim. Sci..

[25] Vassalo F.G., Rahal S.C., Agostinho F.S., Mamprim M.J., Melchert A., Kano W.T., dos Reis Mesquita L., Doiche D.P. (2015). Gait analysis in dogs with pelvic fractures treated conservatively using a pressure-sensing walkway. Acta Vet. Scand..

[26] von der Ahe C., Marahrens H., Schwarze M., Angrisani N., Reifenrath J. (2023). Pressure sensing mat as an objective and sensitive tool for the evaluation of lameness in rabbits. PLoS ONE.

[27] Laflamme D.P. (1997). Development and validation of a body condition score system for dogs. Canine Pract..

[28] Cook J.L., Evans R., Conzemius M.G., Lascelles B.D., McIlwraith C.W., Pozzi A., Clegg P., Innes J., Schulz K., Houlton J. (2010). Proposed definitions and criteria for reporting time frame, outcome, and complications for clinical orthopedic studies in veterinary medicine. Vet. Surg..

[29] Gibert S., Lequang T., Maitre P., Poujol L., Cachon T., Carozzo C., Fau D., Genevois J.P., Viguier E. (2010). Sensitivity and specificity to determine lameness in dogs with a pressure walkway system. Comput. Methods Biomech. Biomed. Eng..

[30] Le Quang T., Maitre P., Roger T., Viguier E. (2007). The GAITRite^®^ system for evaluation of the spatial and temporal parameters of normal dogs at a walk. Comput. Methods Biomech. Biomed. Eng..

[31] Fahie M.A., Cortez J.C., Ledesma M., Su Y. (2018). Pressure Mat Analysis of Walk and Trot Gait Characteristics in 66 Normal Small, Medium, Large, and Giant Breed Dogs. Front. Vet. Sci..

[32] Light V.A., Steiss J.E., Montgomery R.D., Rumph P.F., Wright J.C. (2010). Temporal-spatial gait analysis by use of a portable walkway system in healthy Labrador Retrievers at a walk. Am. J. Vet. Res..

[33] LeQuang T., Maitre P., Colin A., Roger T., Viguier E. Gait Analysis for Sound Dogs at a Walk by Using a Pressure Walkway. Proceedings of the The Third International Conference on the Development of Biomedical Engineering in Vietnam.

[34] Brønniche Møller Nielsen M., Pedersen T., Mouritzen A., Vitger A.D., Nielsen L.N., Poulsen H.H., Miles J.E. (2020). Kinetic gait analysis in healthy dogs and dogs with osteoarthritis: An evaluation of precision and overlap performance of a pressure-sensitive walkway and the use of symmetry indices. PLoS ONE.

[35] Gordon-Evans W.J., Evans R.B., Knap K.E., Hildreth J.M., Pinel C.B., Imhoff D.J., Conzemius M.G. (2009). Characterization of spatiotemporal gait characteristics in clinically normal dogs and dogs with spinal cord disease. Am. J. Vet. Res..

[36] Evans R., Gordon W., Conzemius M. (2003). Effect of velocity on ground reaction forces in dogs with lameness attributable to tearing of the cranial cruciate ligament. Am. J. Vet. Res..

[37] Conzemius M.G., Evans R.B., Besancon M.F., Gordon W.J., Horstman C.L., Hoefle W.D., Nieves M.A., Wagner S.D. (2005). Effect of surgical technique on limb function after surgery for rupture of the cranial cruciate ligament in dogs. J. Am. Vet. Med. Assoc..

[38] Rumph P.F., Kincaid S.A., Visco D.M., Baird D.K., Kammermann J.R., West M.S. (1995). Redistribution of vertical ground reaction force in dogs with experimentally induced chronic hindlimb lameness. Vet. Surg..

[39] Della Valle G., Caterino C., Aragosa F., Micieli F., Costanza D., Di Palma C., Piscitelli A., Fatone G. (2021). Outcome after Modified Maquet Procedure in dogs with unilateral cranial cruciate ligament rupture: Evaluation of recovery limb function by use of force plate gait analysis. PLoS ONE.

[40] Wu S.H., Li Y., Zhang Y.Q., Li X.K., Yuan C.F., Hao Y.L., Zhang Z.Y., Guo Z. (2013). Porous titanium-6 aluminum-4 vanadium cage has better osseointegration and less micromotion than a poly-ether-ether-ketone cage in sheep vertebral fusion. Artif. Organs.

[41] Maitre P., Arnault A., Verset M., Roger T., Viguier E. (2007). Chronic cranial cruciate ligament rupture in dog: Four legs assessment with a walkway. Comput. Methods Biomech. Biomed. Eng..

[42] Voss K., Damur D.M., Guerrero T., Haessig M., Montavon P.M. (2008). Force plate gait analysis to assess limb function after tibial tuberosity advancement in dogs with cranial cruciate ligament disease. Vet. Comp. Orthop. Traumatol..

[43] Lascelles B.D., Roe S.C., Smith E., Reynolds L., Markham J., Marcellin-Little D., Bergh M.S., Budsberg S.C. (2006). Evaluation of a pressure walkway system for measurement of vertical limb forces in clinically normal dogs. Am. J. Vet. Res..

[44] Fanchon L., Grandjean D. (2007). Accuracy of asymmetry indices of ground reaction forces for diagnosis of hind limb lameness in dogs. Am. J. Vet. Res..

[45] Ferreira M.P., Ferrigno C.R., de Souza A.N., Caquias D.F., de Figueiredo A.V. (2016). Short-term comparison of tibial tuberosity advancement and tibial plateau levelling osteotomy in dogs with cranial cruciate ligament disease using kinetic analysis. Vet. Comp. Orthop. Traumatol..

